# Specific and sensitive detection of bovine coronavirus using CRISPR-Cas13a combined with RT-RAA technology

**DOI:** 10.3389/fvets.2024.1473674

**Published:** 2025-01-07

**Authors:** Zili Liang, Ruxing Luo, Qifu He, Cheng Tang, Zhidong Zhang, Yanmin Li, Zijing Guo

**Affiliations:** ^1^Key Laboratory of Animal Medicine at Southwest Minzu University of Sichuan Province, College of Animal Science and Veterinary Medicine, Southwest Minzu University, Chengdu, China; ^2^Wuhou District Health Hospital for Women & Children, Chengdu, China

**Keywords:** CRISPR/Cas13a, RT-RAA, bovine coronavirus, diagnostic, nucleic acid detection

## Abstract

**Introduction:**

Bovine coronavirus (BCoV) is an important pathogen of enteric and respiratory disease in cattle, resulting in huge economic losses to the beef and dairy industries worldwide. A specific and sensitive detection assay for BCoV is critical to the early-stage disease prevention and control.

**Methods:**

We established a specific, sensitive, and stable assay for BCoV nucleic acid detection based on CRISPR/Cas13a combined with reverse transcription recombinase-aided amplification (RT-RAA) technology. The specific primers for RT-RAA and CRISPR RNA (crRNA) were designed in the conserved region of the BCoV nucleocapsid (N) gene.

**Results:**

The detection limit of the RT-RAA CRISPR/Cas13a assays for BCoV detection was 1.72 copies/μl, and there were no cross-reactions with the other 10 common bovine enteric and respiratory disease-associated pathogens. The coefficient of variations (CVs) of within and between batches were less than 4.98 and 4.58%, respectively. The RT-RAA-CRISPR/Cas13a assays work well in clinical samples of cattle and yak, the BCoV positive rate of 84 clinical samples detected by RT-RAA-CRISPR/Cas13a assays was 58.3% (49/84), it was notably higher than that of RT-qPCR (2.4%, 2/84; *p* < 0.001). The 49 positive samples detected by RT-RAA-CRISPR/Cas13a assays were further confirmed as BCoV by Sanger sequencing.

**Discussion:**

A specific, sensitive, and stable assay based on RT-RAA-CRISPR/Cas13a assays for BCoV was developed, providing new technical support for the clinical detection and epidemiological monitoring of BCoV.

## 1 Introduction

Bovine coronavirus (BCoV) belongs to the genus *Betacoronavirus* of the family *Coronaviridae*, with a single-stranded and positive-sense RNA genome.^1^ BCoV can cause bovine respiratory disease, neonatal calf diarrhea, winter dysentery, and shipping fever in adult cattle (1). These illnesses can cause weight loss, dehydration, decreased milk production, and even death. Until now, BCoV has a wide epidemic range involving at least 25 countries from North America, Asia, Europe, Oceania, and Africa (2). Moreover, BCoV has been found in the clinical samples from humans, sheep, musk, oxen, elk, sambar, deer, goat, dromedary, camel, alpaca, giraffe, and wisent, indicating that BCoV may has the potential for cross-species transmission (2). Taken together, BCoV not only exhibited serious harm for the healthy development of cattle industry, but also posed a potential threat to global public health.

At present, there are many detection methods for BCoV, including direct electron microscopy, serological neutralization test, immunohistochemical method (3), enzyme-linked immunosorbent assay (4), polymerase chain reaction (PCR) (5, 6), isothermal amplification-based techniques (7), and clustered regularly interspaced short palindromic repeats (CRISPR)/Cas13a-based assay (8). A variety of PCR techniques have been established for BCoV detection, including reverse transcription-PCR (RT-PCR) (9), semi-nested PCR assay (10), real-time RT-PCR (RT-qPCR), multiplex RT-qPCR (11), and multiplex droplet digital PCR(ddPCR) (12). At present, RT-PCR and RT-qPCR were widely selected for BCoV detection and its epidemiological investigation (11, 13, 14). These assays play a vital role in the detection of BCoV infection, but these methods for BCoV detection require complex laboratory equipment and expensive temperature-controlled amplification devices.

CRISPR-associated systems (Cas) exhibit great potential in the fields of RNA interference and gene editing (15). Among them, CRISPR-related proteins Cas13a can specifically cut the target RNA under the guidance of crRNA and remain active after the cutting is completed, continuing to cut other non-target RNA, which is known as the “collateral effect” (16). Based on this principle, CRISPR/Cas13 can detect the RNA template and amplify the signal to achieve high specific detection of the target molecule by designing RNA probes labeled with fluorophores at both ends (17). Reverse transcription recombinase-aided amplification (RT-RAA) is a technique for nucleic acid amplification within 10 ~ 30 min under the isothermal condition (18). Recently, a practical molecular diagnostic technology platform called SHERLOCK with ultrasensitivity and high specificity was developed based on CRISPR/Cas technology combined with isothermal amplification (16). This technology exhibited incomparable advantages compared to the RT-qPCR, and it has been widely used to detect many viruses (19), including severe acute respiratory syndrome coronavirus 2 (SARS-CoV-2) (20), hepatitis B virus (21), porcine reproductive and respiratory syndrome virus (PRRSV) (22), avian influenza virus (AIV) (23), and bovine viral diarrhea virus (BVDV) (24).

In this study, the 52 partial N genes of BCoV from cattle and 40 partial N genes of yak coronavirus in the GenBank database were analyzed, and the highly conserved region was selected to design specific RT-RAA primers and crRNA. The important three components are RNA-FQ reporter, crRNA, and Cas13a, which were sequentially optimized. The sensitivity, specificity, and stability of this assay were evaluated. This study aims to establish a specific, sensitive, and stable detection for BCoV nucleic acid based on CRISPR/Cas13a combined with RT-RAA technology, providing an alternative tool for the detection of BCoV.

## 2 Materials and methods

### 2.1 Virus and clinical samples

The BCoV strain isolated from a diarrhea fecal sample of a calf was stored in the Key Laboratory of Animal Medicine at Southwest Minzu University. The nucleic acid of bovine enteric and respiratory disease associated-pathogens were used to evaluate the specificity and were stored in Key Laboratory of Animal Medicine at Southwest Minzu University, including bovine rotavirus (BRV), BVDV, bovine enterovirus (BEV), bovine astrovirus (BAstV), infectious bovine rhinotracheitis virus (IBRV), bovine respiratory syncytial virus (BRSV), bovine parainfluenza virus 3 (BPIV3), bovine *Escherichia coli* K99, bovine Salmonella Dublin, and *Mycoplasma bovis*. In 2023, 50 clinical samples of beef cattle (28 diarrhea fecal samples, 22 nasal swabs from calves with bovine respiratory disease symptoms) were collected in Sichuan and Jilin Provinces, China. 34 clinical samples of yak (20 diarrhea fecal samples, 14 nasal swabs from yak with bovine respiratory disease symptoms) were collected in Sichuan Province in 2023. All samples were stored at −80°C.

### 2.2 Nucleic acid extraction, cDNA, and plasmid preparation

The DNA or RNA of clinical samples was extracted using the AxyGen Nucleic Acid Extraction Kit (Geneaid, China, Shanghai) according to the manufacturer’s instruction, and the DNA or RNA was eluted in nuclease-free water. The total RNA was reverse transcribed into cDNA using the Prime Script RT Master Mix (Takara, China, Chengdu) according to the manufacturer’s instructions. The plasmid containing 738 bp BCoV N gene sequence (located at nt 29,390 ~ 30,127 in the reference genome of BCoV/SWUN/HXD1/2022, GenBank accession number: OR612022.1) was constructed using pMD19-T simple vector (TaKaRa, China, Chengdu.) and sequenced by Sangon Biotech, Co., LTD, China.

### 2.3 Design of primers, probes, and CrRNA preparation

The 52 all available partial N gene of BCoV from cattle and the 40 all available partial N gene of yak coronavirus in the GenBank database were analyzed by MEGA11.0 software (25), and the 92 BCoV strains information were shown in Supplementary Table 1. The partial N gene of the BCoV was located from 29,522 nt to 30,163 nt of the referenced BCoV-EN strain (GenBank accession number. NC_003045.1), and the highly conserved region was selected to design specific CRISPR-RNA (crRNA) and RT-RAA primers containing T7 promoter by RPA/RAA primer designer online software of ezassay company^2^ and BLAST.^3^ crRNA was synthesized by Guangzhou Bolis Biotechnology Co., LTD, China. The fluorescence RNA reporter is RNA with 5′modified to 6-FAM, 3′modified to BHQ-1. The designed primers and reporter oligonucleotides were synthesized by Sangon Biotech, China. All sequences used in this study were shown in Table 1.

**Table 1:** Sequence of primers, crRNA and reporter in this study.

Name	Sequence (5′ → 3′)
RAA-F	ACTGGTACAGACACAACAGACGTTCCTTTAA
T7-RAA-F	**GAAATTAATACGACTCACTATAGGG** ACTGGTACAGACACAACAGACG
RAA-R1	AGACTCCGTCAATGTCGGTGCCATACTGGTC
RAA-R2	GCGACCCAGAAGACTCCGTCAATGTCGGTGC
RAA-R3	GTTACTAGCGACCCAGAAGACTCCGTCAATG
CrRNA	GAUUUAGACUACCCCAAAAACGAAGGGGACUAAAACAUAGUAAAAAUACCAUCGUGGCAGCAAU
Reporter	6-FAM-UUUUUUUUUUUUUU-BHQ1
BCoV-738-F	AGAGCGTCCTCTGGAAATCG
BCOV-738-R	CTGCTTAGTTACTTGCTGTGGCT

### 2.4 Reverse-transcription recombinase-aided amplification

Isothermal amplification was performed according to the instructions of the RT-RAA amplification kit (basic type; ZC Bio-Sci&Tech, China, Hangzhou). For the reaction system, 13.5 μL of nuclease-free water, 25 μL of Buffer A (10% polyethylene glycol), and 2 μL of upstream and downstream primers (10 nM) were combined uniformly into the detecting unit tube containing dry reaction powder. Then, a 5 μL DNA or RNA sample was placed into the detection unit tube, 2.5 μL Buffer B (Mg^2+^ buffer solution) was then added to the detection unit’s tube cover, the tube cover was closed, and the mixture was completely mixed 5 ~ 6 times with gentle shaking upside down, centrifuged at low speed for 10 s, and put in a water bath at 40°C for 30 min to acquire the amplified products. The amplified products were purified using the FastPure Gel DNA Extraction Kit (Vazyme, China, Nanjing).

### 2.5 Establishment of BCoV CRISPR/Cas13a assay combined RT-RAA

The BCoV-Cas13a assays combined RT-RAA reaction and LwCas13a trans-cleavage system containing T7 transcription were established. For RT-RAA, 5 μL plasmid DNA was amplified in a 50 μL reaction system for 30 min at 40°C, according to the instruction of the RT-RAA amplification kit (basic type). The optimal RT-RAA primer pairs were determined based on the observation of RT-RAA amplicons with a multicolor fluorescence GelView 5000Plus (Boluteng Biotechnology Co., Ltd., China, Guangzhou) and the nucleic acid concentration of the RT-RAA products with an ultramicro spectrophotometer (Kaiao Technology Development Co., Ltd., China, Beijing). The 25 μL BCoV-Cas13a reaction system that consisted of 1 μL RT-RAA amplicon, 50 ~ 200 nM LwCas13a protein (Magigen, China, Guangzhou), 30 ~ 90 nM crRNA, 1.6 U/μl Rnase inhibitor (New England Biolabs, United States), 0.5 ~ 2.0 μM reporter, 2 μL T7 RNA Polymerase (New England Biolabs, United States), 2 μL RNAPol reaction buffer (New England Biolabs, United States), 0.4 μL NTP Buffer Mix (New England Biolabs, United States) and supplementary Dnase/Rnase-free water (Solarbio, China, Beijing) was incubated for 30 min at 37°C. The fluorescence value was determined by the Real-time PCR System Archimed X4 (Roc Gene Scientific Instrument Co., LTD, China, Beijing), and the visual readouts were observed by a multicolor fluorescence GelView 5000Plus and blue Light glue cutter (BluPAD, Bio-Helix, China, Shanghai).

### 2.6 Sensitivity and specificity of RT-RAA-CRISPR/Cas13a assays of BCoV

For sensitivity testing, the serially diluted plasmid DNA templates (10^8^ ~ 10^−2^ copies/μl) were prepared and were detected by the RT-RAA-CRISPR/Cas13a assays of BCoV. The specificity of RT-RAA-CRISPR/Cas13a assays of BCoV was verified by detecting nucleic acids of bovine enteric and respiratory disease-associated pathogens (BRV, BVDV, BEV, BAstV, IBRV, BRSV, BPIV3, bovine *Escherichia coli* K99, bovine Salmonella Dublin, and *Mycoplasma bovis*.).

### 2.7 Comparison of RT-RAA-CRISPR/Cas13a assays and RT-qPCR for BCoV detection

Extracted nucleic acids from 84 clinical samples were detected using RT-RAA-CRISPR/Cas13a assays and RT-qPCR assay (5). All specimens with a Ct value of ≤40 were considered positive by the RT-qPCR test. For the positive samples determined by RT-RAA-CRISPR/Cas13a assays, the productions of RT-RAA amplicons were purified using the FastPure Gel DNA Extraction Kit (Vazyme, China, Nanjing), and further were Sanger sequenced by Sangon Biotech Co., LTD, China. Nucleotide was analyzed using the MegAlign program of DNASTAR7.0 software (DNASTAR Inc., WI, United States) to determine sequence homology.

### 2.8 Image and statistical data analysis

All data in this study for drawing fluorescence plottings were analyzed using GraphPad Prism 9.8 software. The comparison between RT-RAA-CRISPR/Cas13a assays and the RT-qPCR assay was analyzed using SPSS 16.0 software and the Chi-square test.

## 3 Results

### 3.1 Screening of the optimal RT-RAA primers of BCoV

**Figure 1:**
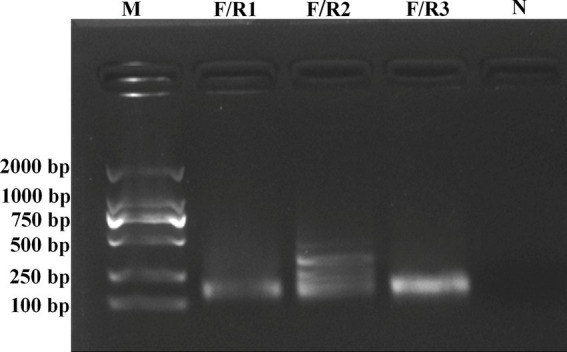
Electrophoretic agarose gels of RT-RAA products amplified by RT-RAA-F/R1, RT-RAA-F/R2, and RT-RAA-F/R3 primers. Lane M represented standard molecular weight DL2000 DNA ladder (Biosharp, China), and Lane N represented negative control (RNase-Free distilled water).

A forward primer (RT-RAA-F) and three reverse primers (RT-RAA-R1, RT-RAA-R2, and RT-RAA-R3) were designed to match the conserved N gene of BCoV. The RT-RAA reactions using 10^3^ copies/μl of plasmid DNA were performed to compare three combinations of the upstream and downstream primers. The results showed that the amplification of RT-RAA-F/R1 generated a single targeted band at about 138 base pair (bp) on an agarose gel, and the nucleic acid concentration of the RT-RAA products by RT-RAA-F/R1 primers was 22.3 ng/μl. For RT-RAA-F/R2 primers, the amplifications showed many non-specific bands (targeted band at 148 bp). However, the amplification of RT-RAA-F/R3 also amplified a single specific band at about 155 bp, which was consistent with the expected fragment size. The nucleic acid concentration of RT-RAA products by RT-RAA-F/R3 primers was 56.3 ng/μl, which is higher than that of the products by RT-RAA-F/R1 primers. No band was observed in the negative control (nuclease-free water was used as the template). Therefore, the RT-RAA-F/R3 primer pair was selected for further experiments (Figure 1).

### 3.2 Establishment and optimization of RT-RAA-CRISPR/Cas13a assays of BCoV

**Figure 2:**
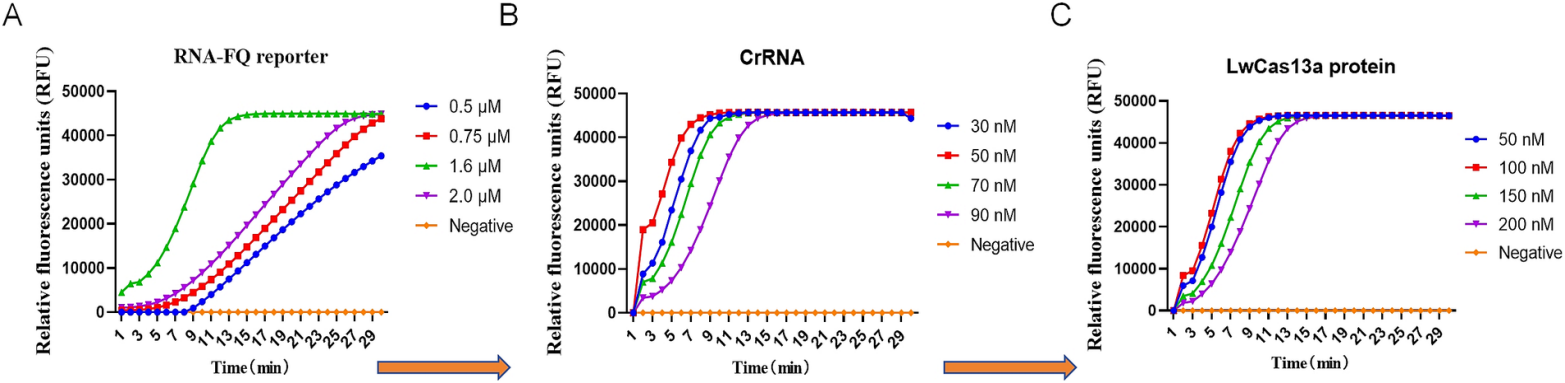
Establishment and optimization of RT-RAA-CRISPR/Cas13a assays of BCoV. **(A)** The RNA-FQ reporter (0.50 μM, 0.75 μM, 1.60 μM, 2.0 μM) for the RT-RAA-CRISPR/Cas13a assays of BCoV was optimized based on the relative fluorescence units. **(B)** The crRNA (30 nM, 50 nM, 70 nM, 90 nM) for the RT-RAA-CRISPR/Cas13a assays of BCoV was optimized based on the relative fluorescence units. **(C)** LwCas13a (50 nM, 100 nM, 150 nM, 200 nM) for the RT-RAA-CRISPR/Cas13a assays of BCoV was optimized based on the relative fluorescence units.

The reaction component of the CRISPR detection assay was further optimized using plasmid DNA (10^3^ copies/μl) as a positive template. The RNA-FQ reporter (0.50 μM, 0.75 μM, 1.60 μM, 2.0 μM), crRNA (30 nM, 50 nM, 70 nM, 90 nM), and Cas13a (50 nM, 100 nM, 150 nM, 200 nM) were sequentially optimized by fixing the concentration of the other components. The results showed that the optimal concentration of RNA-FQ reporter, crRNA, and Cas13a were 1.6 μM (0.64 μL), 70 nM (1.40 μL), and 100 nM (1.00 μL), respectively, as shown in Figure 2. The final version of 20 μL reaction system consisted of 1 μL RAA amplicons, 100 nM Cas13a, 70 nM crRNA, 20 Units RNase inhibitor (0.50 μL), 1.60 μM FQ-reporter, 2 μL T7 RNA polymerase, 2 μL RNAPol reaction buffer, 0.40 μL NTP buffer mix, and reaction buffer was added to 20 μL. The fluorescence value could be determined within 30 min at 37°C.

### 3.3 Sensitivity of RT-RAA-CRISPR/Cas13a assays of BCoV

**Figure 3:**
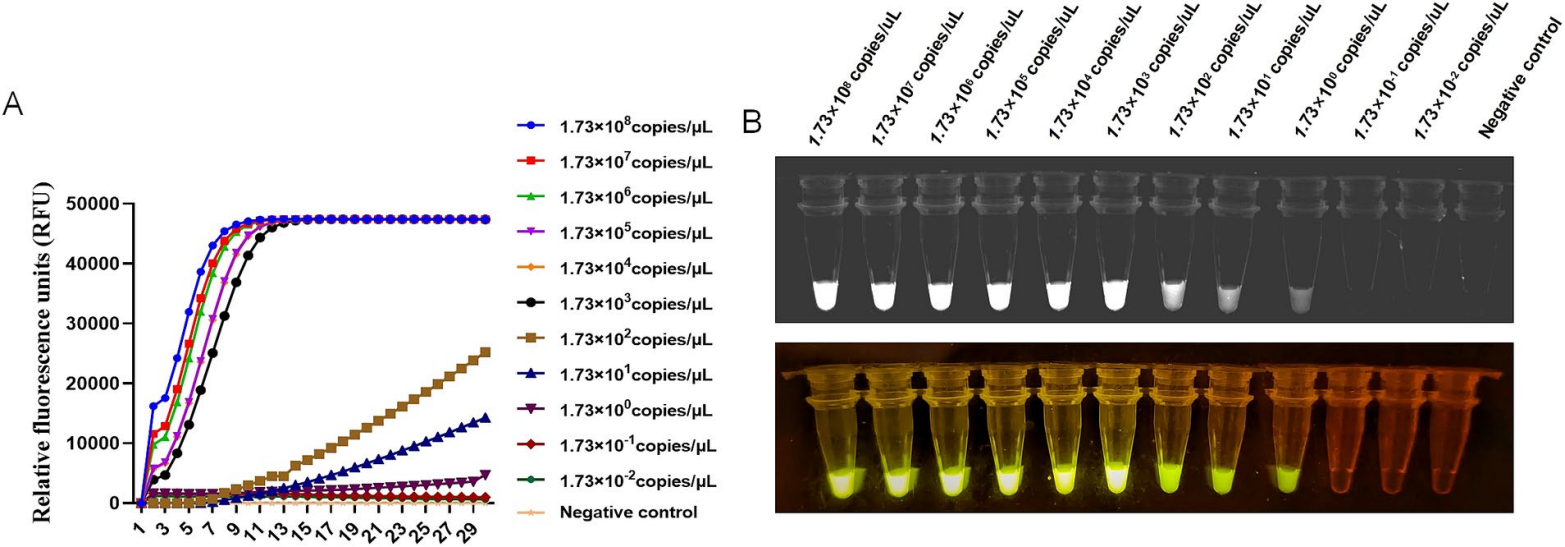
Sensitivity of RT-RAA-CRISPR/Cas13a assays of BCoV. **(A)** Relative Fluorescence units of RT-RAA-CRISPR/Cas13a assays for 12 different gradients of 10^8^ copies/μL to 10^−3^ copies/μL BCoV plasmid were read by the Real-time PCR System. **(B)** The RT-RAA-CRISPR/Cas13a assays productions of 12 different gradients were observed by a multicolor fluorescence GelView 5000Plus and blue Light glue cutter. Negative control (RNase-Free distilled water).

To evaluate the sensitivity of CRISPR-Cas13a-based detection of BCoV, the standard plasmid was diluted in 10-fold gradient using DNase/RNase-free water. The initial concentration of plasmid was 65 ng/μl containing 1.73 × 10^10^ copies/μL. The fluorescence values of the 12 detection gradients from 10^8^ copies/μL to 10^−3^ copies/μL were tested, shown in Figure 3. The fluorescent signal could be detected in the 10^0^ dilution, which indicated that the limit of detection using fluorescent readout was below 1.73 copies/μL (Figure 3).

### 3.4 Specificity of RT-RAA-CRISPR/Cas13a assays of BCoV

**Figure 4:**
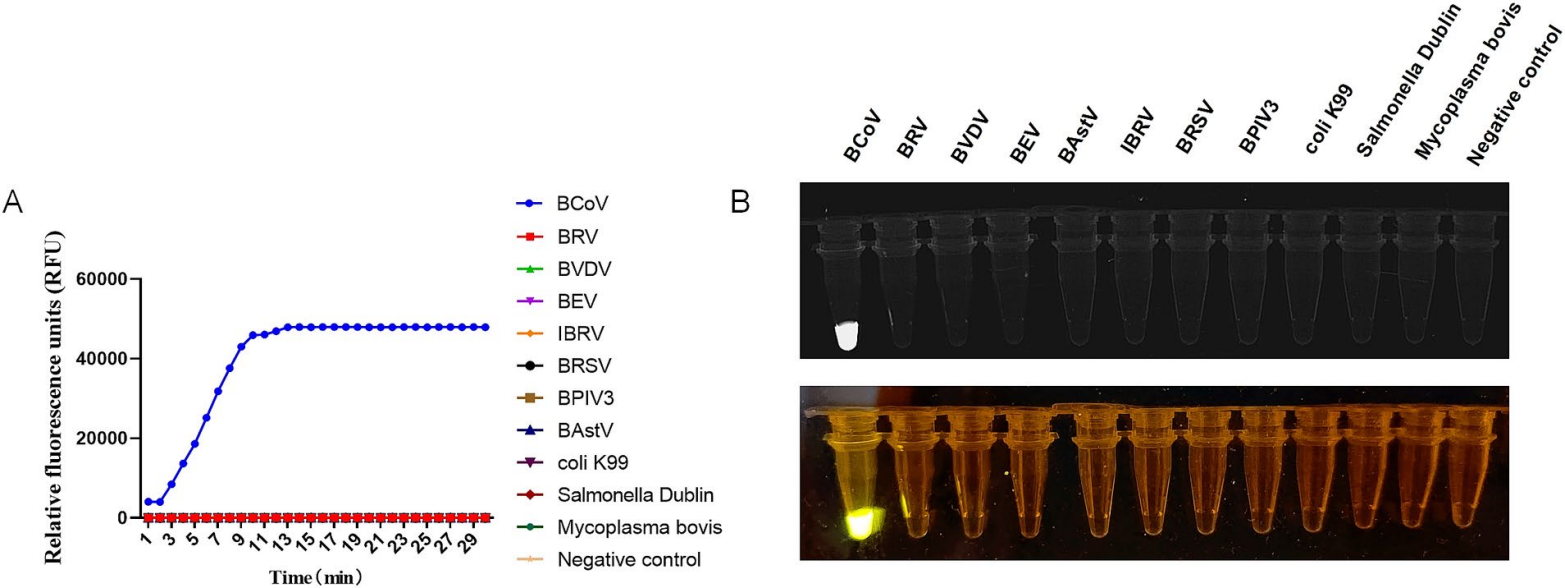
Specificity of RT-RAA-CRISPR/Cas13a assays of BCoV. **(A)** Relative Fluorescence units of RT-RAA-CRISPR/Cas13a assays for BCoV and other 10 common bovine enteric and respiratory disease associated pathogens. **(B)** The RT-RAA-CRISPR/Cas13a assays productions of BCoV and other 10 common bovine enteric and respiratory disease associated pathogens were observed by a multicolor fluorescence GelView 5000Plus and blue Light glue cutter. Negative control (RNase-Free distilled water).

The specificity of CRISPR-Cas13a-based detection of BCoV was analyzed using other common bovine enteric and respiratory disease-associated pathogens (BRV, BVDV, BEV, BAstV, IBRV, BRSV, BPIV3, bovine *Escherichia coli* K99, bovine Salmonella Dublin, and *Mycoplasma bovis*). The fluorescence kinetics showed that BCoV alone produced fluorescence signals, whereas all other samples did not exhibit fluorescence (Figure 4). These data demonstrated that the established CRISPR-Cas13a-based detection method was specific for detecting BCoV.

### 3.5 Repeatability of RT-RAA-CRISPR/Cas13a assays of BCoV

The repeated tests within and between batches were performed using three concentrations: 1.73 × 10^8^ copies/μL (high concentration), 1.73 × 10^5^ copies/μL (medium concentration), and 1.73 × 10^2^ copies/μL (low concentration) of BCoV plasmid DNA. The intra-batch tests were performed in three repeats with one run, while the inter-batch tests were conducted by three independent runs within 3 days. The coefficient of variations (CVs) of repeated tests intra-batch and inter-batch were determined by calculating the fluorescence values. As shown in Table 2, the CVs within and between batches were less than 4.98 and 4.58%, respectively, suggesting that the established method was well stable.

**Table 2:** Repeated tests within and between batches of the RT-RAA-CRISPR/Cas13a assays.

BCoV nucleic acid	Repeatability (Intra-batch test)			Reproducibility (Inter-batch test)		
concentration(copies/uL)	Mean	SD	CV	Mean	SD	CV
High (1.73*10^8^)	48245.99	1698.73	3.52%	51292.05	1735.03	3.38%
Medium (1.73*10^5^)	44,861	#1339.06	0.76%	49732.77	2413.27	4.58%
Low (1.73*10^2^)	40003.14	1993.27	4.98%	39258.55	1,555	1.41%

### 3.6 Clinical detection by RT-RAA-CRISPR/Cas13a assays and RT-qPCR

**Figure 5:**
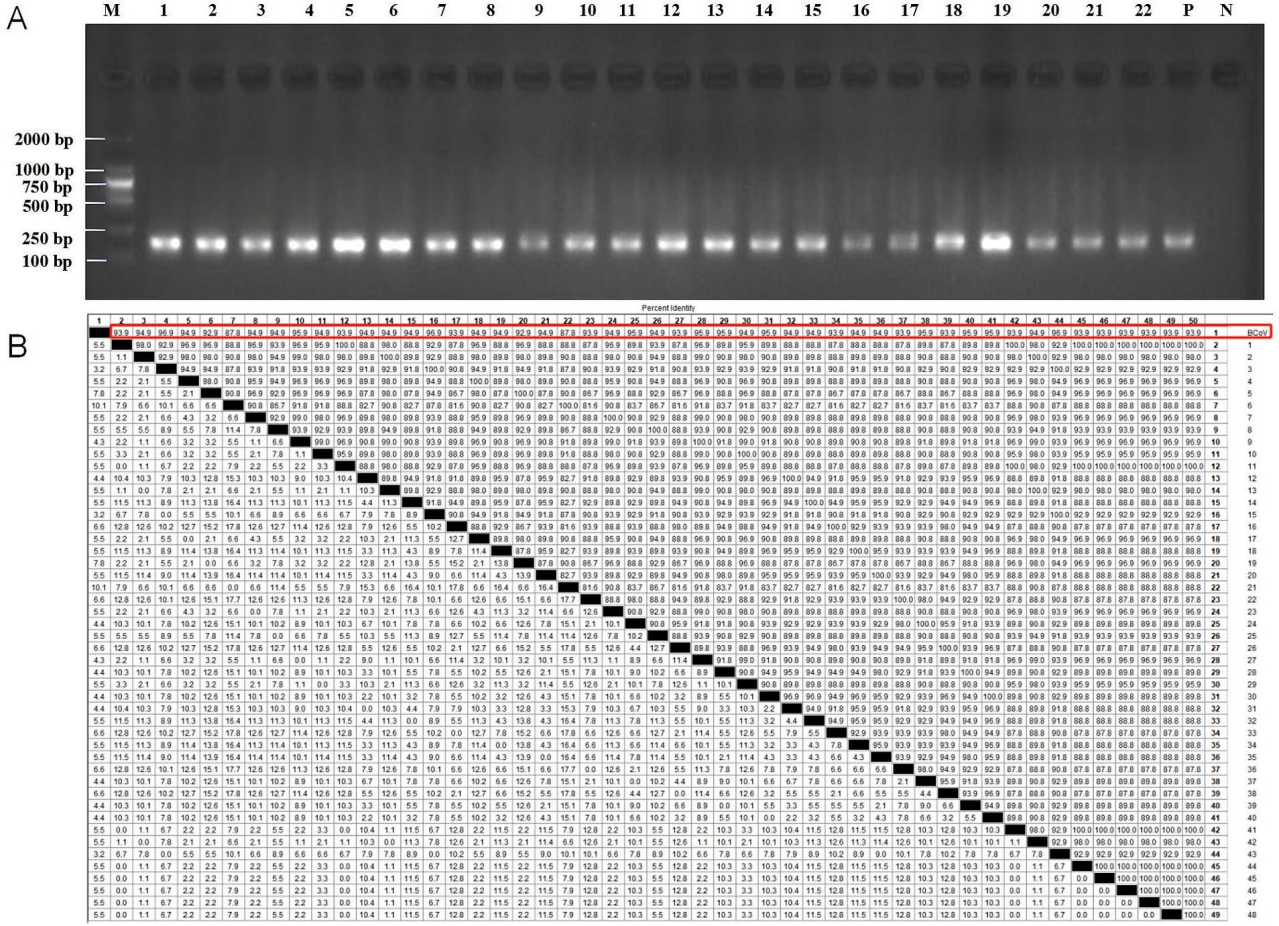
All positive samples detected by RT-RAA-CRISPR/Cas13a assays were further verified by RT-RAA amplicon-DNA sequencing. **(A)** Electrophoretic agarose gels of the RT-RAA-amplified products of the 22 partial positive samples. M: D2000 DNA ladder (Biotopped, China), lanes 1–22 were amplified products of partial positive samples, P: positive control (10^3^ copies/μl of plasmid DNA), N: negative control (RNase-Free distilled water). **(B)** The nucleotide similarity of 49 BCoV strains in this study and BCoV referenced strain in the GenBank database. The red box showed that 49 BCoV strains in this study shared nucleotide similarity with the BCoV-referenced strain (BCoV-China/SWUN/A1/2018).

The RT-RAA-CRISPR/Cas13a assays established in this study was used to detect BCoV in 48 diarrhea stool samples of calves and 36 nasal swabs of calves with respiratory syndrome collected from Jilin and Sichuan provinces in 2023. The positive rate of RT-RAA-CRISPR/Cas13a assays was 58.3% (49/84), which was significantly higher than that of RT-qPCR (2.4%, 2/84; *p* < 0.001; Table 3). The positive samples assessed by RT-RAA-CRISPR/Cas13a assays coincided with the positive samples assessed by RT-qPCR. However, all positive samples detected by RT-RAA-CRISPR/Cas13a assays were further confirmed as BCoV by RT-RAA amplicon-DNA sequencing. Specifically, the productions of RT-RAA amplicons were purified and were observed by electrophoretic agarose gels (Figure 5A; Supplementary Figure 3). These amplified fragments were further Sanger sequenced. Sequence analysis showed that the 49 BCoV strains in this study shared 87.8% ~ 96.9.0% nucleotide identity with BCoV referenced strain (BCoV-China/SWUN/A1/2018) in the GenBank database (Figure 5B), indicating that the 49 clinical samples were positive for BCoV. It demonstrated the accuracy of the detection results of the RT-RAA-CRISPR/Cas13a assays established in this study.

**Table 3:** Detection in clinical samples by RT-RAA-CRISPR/Cas13a assays and RT-qPCR assay.

Test method	Beef cattle		Yak	
	Diarrhea stool	Nasal swabs	Diarrhea stool	Nasal swabs
RT-qPCR (5)	11.11% (2/28)	0.00% (0/22)	0.00% (0/20)	0.00% (0/14)
This study	67.86% (19/28)	50.00% (11/22)	75.00% (15/20)	28.57% (4/14)

## 4 Discussion

BCoV can result in neonatal calf diarrhea, winter dysentery, and respiratory diseases in adult cattle (1). It is still widespread in the world and is one of the important viral diseases endangering the cattle industry (2). An efficient, sensitive, and specific detection assay for BCoV is important for the early-stage disease prevention and control of BCoV. This study combines RT-RAA technology with the CRISPR/Cas13a system to realize the complementary advantages of the two technologies, CRISPR-Cas13a eliminates non-specific amplification signals from RT-RAA, while RT-RAA enhances the sensitivity of CRISPR-Cas system (16). The RT-RAA-CRISPR/Cas13a assays of BCoV established in this study was highly sensitive, specific, quick, and repeatable (16). In addition, the test result was visual via observing the fluorescence signal with a small light illuminator under the LED blue. This assay provided a novel strategy for the detection of BCoV. To further facilitate BCoV detection of this assay in the field, the process of nucleic acid extraction needs to be further addressed. Many simpler and instrument-free nucleic acid extraction techniques have been developed (26). Recent a study showed that the SARS-CoV-2 RNA genome was extracted from an entire nasopharyngeal or anterior nasal swab via combining the lysis and magnetic bead–binding steps, and further used in SHERLOCK testing (27). In addition, shortening the detection time and reducing the cost need to be considered in future studies.

For molecular detection methods of pathogen, the nucleotide variation of detection targets may lead to low amplification efficiency or amplification failure. Studies suggested that the N gene (7), M gene (5, 28), and Nsp10 (9) of BCoV were relatively conserved, thus they were often used as target genes for BCoV molecular detection. In this study, all 92 available BCoV N gene sequences were analyzed, the primers of RT-RAA and CrRNA were designed in the conserved N gene region, and the RT-RAA-CRISPR/Cas13a assays for BCoV detection was established. The detection rate of this assay for clinical samples (49/84) was significantly higher than that of RT-qPCR (2/84). The 49 positive samples detected by RT-RAA-CRISPR/Cas13a assays were further confirmed as BCoV by Sanger sequencing. Sequence analysis of 38 available M genes of BCoV in the GenBank database showed that the current circulating strains have 7 nucleotide mutations and 14 nucleotide mutations in the reverse primer (18 nt) and probe sequence (28 nt) of RT-qPCR, respectively (Supplementary Figure 1). These nucleotide mutations could result in mismatches between amplification primers and target sequences. In addition, the detected limit of the RT-qPCR (20 copies/uL) was higher than that of RT-RAA-CRISPR/Cas13a assays (1.73 copies/uL) (5). These factors could cause a false negative in a sample, resulting in a significantly high detection rate of BCoV by the RT-RAA-CRISPR/Cas13a assays.

The yak (*Bosgrunniens*) is a unique domestic bovine species that plays an indispensable role for herdsmen in the Qinghai-Tibet Plateau (29). Our previous study found that BCoV strains from yak showed many nucleotide mutations in the N gene, spike (S), and haemagglutinin-esterase (HE) gene (30), which may lead to a false negative for BCoV sample. Considering the unique evolution of BCoV strains from yak, the primer design was also based on the available partial N genes sequence of 40 BCoV strains of yak in the GenBank database. This study showed that the approximately 60.00% (30/50) samples collected from beef cattle and 52.94% (18/34) samples collected from yak were assessed as BCoV-positive by RT-RAA-CRISPR/Cas13a assays. The result suggested that the RT-RAA-CRISPR/Cas13a assays were not only applied to the respiratory tract and fecal samples but also had a good detection effect on clinical samples in beef cattle and yak. Taken together, the RT-RAA-CRISPR/Cas13a assays provided a powerful tool for the detection and epidemiological investigation of BCoV in beef cattle and yak.

## 5 Conclusion

In this study, a specific, sensitive, and stable assay based on the RT-RAA-CRISPR/Cas13a assays was established for rapid detection of BCoV nucleic acid in clinical samples from beef cattle and yak, which could be applied to the diagnosis, monitoring, and epidemiological investigation of BCoV. This assay provided technical support for the effective prevention and control of bovine diarrhea and respiratory disease caused by BCoV.

## Data Availability

The original contributions presented in the study are included in the article/Supplementary material, further inquiries can be directed to the corresponding author.

## Footnotes

^1^ https://talk.ictvonline.org/

^2^ https://www.ezassay.com/primer

^3^ https://blast.ncbi.nlm.nih.gov/
