# Supplementary material for: Specific and sensitive detection of bovine coronavirus using CRISPR-Cas13a combined with RT-RAA technology
Source: Front Vet Sci. 2025 Jan 7;11:1473674. doi: 10.3389/fvets.2024.1473674 (PMC11749252; doi:10.3389/fvets.2024.1473674)
Supplement: Supplementary file 1 [file Table_1.DOCX]

**Supplementary Table 1**. Reference strains were used to design primers and crRNA in this study.

| Strain | Year | Host | Country | Accession number |
| --- | --- | --- | --- | --- |
| B277a/2021 | 2022 | Dairy Calves | China | OP866729.1 |
| B298/2021 | 2022 | Dairy Calves | China | OP866728.1 |
| F226/2021 | 2022 | Dairy Calves | China | OP866727.1 |
| B277b/2021 | 2022 | Dairy Calves | China | OP866726.1 |
| NMG1/2022 | 2022 | Bovine | China | OP924545.1 |
| EPI/Caen/2004/14 | 2016 | Bovine | France | KT318096.1 |
| FRA/EPI/Caen/2014/13 | 2016 | Bovine | France | KT318095.1 |
| FRA/EPI/Caen/2014/12 | 2016 | Bovine | France | KT318094.1 |
| FRA/EPI/Caen/2013/11 | 2016 | Bovine | France | KT318093.1 |
| FRA/EPI/Caen/2013/10 | 2016 | Bovine | France | KT318092.1 |
| FRA/EPI/Caen/2013/09 | 2016 | Bovine | France | KT318091.1 |
| FRA/EPI/Caen/2013/08 | 2016 | Bovine | France | KT318090.1 |
| FRA/EPI/Caen/2012/07 | 2016 | Bovine | France | KT318089.1 |
| FRA/EPI/Caen/2010/06 | 2016 | Bovine | France | KT318088 |
| FRA/EPI/Caen/2008/04 | 2016 | Bovine | France | KT318086.1 |
| FRA/EPI/Caen/2007/03 | 2016 | Bovine | France | KT318085.1 |
| FRA/EPI/Caen/2005/01 | 2016 | Bovine | France | KT318084.1 |
| FRA/EPI/Caen/2005/02 | 2016 | Bovine | France | KT318083.1 |
| IND/2022/N471 | 2023 | Bos indicus | India | P820541.1 |
| IND/2022/N388 | 2023 | Bos indicus | India | OP820540.1 |
| IND/2021/N77 | 2023 | Bos indicus | India | OP820539.1 |
| IND/2021/N70 | 2023 | Bubalus bubalis | India | OP820538.1 |
| IND/2021/N61 | 2023 | Bubalus bubalis | India | OP820537.1 |
| IND/2021/N60 | 2023 | Bubalus bubalis | India | OP820536.1 |
| IND/2021/N55 | 2023 | Bubalus bubalis | India | OP820535.1 |
| IND/2021/N48 | 2023 | Bubalus bubalis | India | OP820534.1 |
| IND/2021/N156 | 2023 | Bubalus bubalis | India | OP820533.1 |
| IND/2021/F93 | 2023 | Bubalus bubalis | India | OP820532.1 |
| IND/2021/F85 | 2023 | Bubalus bubalis | India | OP820531.1 |
| IND/2021/F31 | 2023 | Bubalus bubalis | India | OP820530.1 |
| CH/GS-1/2019 | 2020 | Bos taurus | China | MN894884.1 |
| CH/HB-BD/2019 | 2019 | Cattle | China | MK903505.1 |
| V270 | 2007 | Cattle | Germany | EF193074.1 |
| L9 | 2007 | Cattle | Germany | EF193073.1 |
| BCoV_2014_13 | 2017 | Bos taurus | France | KX982264.1 |
| BCoV/NX-1/CHN/2022 | 2024 | Cattle | China | OR947456.1 |
| BCoV-ENT | 2020 | Cattle | USA | NC_003045.1 |
| BCoV-LUN | 2002 | Cattle | USA | AF391542.1 |
| BCoV-ENT | 2002 | Cattle | USA | AF391541.1 |
| BCOV-China/SWUN/HN3/2018 | 2019 | Bos taurus | China | MK095173.1 |
| BCOV-China/SWUN/HN2/2018 | 2019 | Bos taurus | China | MK095172.1 |
| BCOV-China/SWUN/HN1/2018 | 2019 | Bos taurus | China | MK095171.1 |
| BCOV-China/SWUN/LN5/2018 | 2019 | Bos taurus | China | MK095170.1 |
| BCOV-China/SWUN/LN4/2018 | 2019 | Bos taurus | China | MK095169.1 |
| BCOV-China/SWUN/LN3/2018 | 2019 | Bos taurus | China | MK095168.1 |
| BCOV-China/SWUN/LN2/2018 | 2019 | Bos taurus | China | MK095167.1 |
| BCOV-China/SWUN/LN1/2018 | 2019 | Bos taurus | China | MK095166.1 |
| BCOV-China/SWUN/SX2/2018 | 2019 | Bos taurus | China | MK095165.1 |
| BCOV-China/SWUN/ SX1/2018 | 2019 | Bos taurus | China | MK095164.1 |
| BCOV-China/SWUN/SC3/2017 | 2019 | Bos taurus | China | MK095163.1 |
| BCOV-China/SWUN/SC2/2017 | 2019 | Bos taurus | China | MK095162.1 |
| BCOV-China/SWUN/SC1/2017 | 2019 | Bos taurus | China | MK095161.1 |
| QH1 | 2019 | Yak | China | MH741383.1 |
| QH2 | 2019 | Yak | China | MH741382.1 |
| QH3 | 2019 | Yak | China | MH741381.1 |
| QH4 | 2019 | Yak | China | MH741380.1 |
| QH5 | 2019 | Yak | China | MH741379.1 |
| QH6 | 2019 | Yak | China | MH741378.1 |
| QH7 | 2019 | Yak | China | MH741377.1 |
| QH8 | 2019 | Yak | China | MH741376.1 |
| QH9 | 2019 | Yak | China | MH741375.1 |
| QH10 | 2019 | Yak | China | MH741374.1 |
| SC1 | 2019 | Yak | China | MH741373.1 |
| SC2 | 2019 | Yak | China | MH741372.1 |
| SC3 | 2019 | Yak | China | MH741371.1 |
| SC4 | 2019 | Yak | China | MH741370.1 |
| SC5 | 2019 | Yak | China | MH741369.1 |
| SC6 | 2019 | Yak | China | MH741368.1 |
| SC7 | 2019 | Yak | China | MH741367.1 |
| SC8 | 2019 | Yak | China | MH741366.1 |
| SC9 | 2019 | Yak | China | MH741365.1 |
| SC10 | 2019 | Yak | China | MH741364.1 |
| XZ1 | 2019 | Yak | China | MH741363.1 |
| XZ2 | 2019 | Yak | China | MH741362.1 |
| XZ3 | 2019 | Yak | China | MH741361.1 |
| XZ4 | 2019 | Yak | China | MH741360.1 |
| XZ5 | 2019 | Yak | China | MH741359.1 |
| XZ6 | 2019 | Yak | China | MH741358.1 |
| XZ7 | 2019 | Yak | China | MH741357.1 |
| XZ8 | 2019 | Yak | China | MH741356.1 |
| XZ9 | 2019 | Yak | China | MH741355.1 |
| XZ10 | 2019 | Yak | China | MH741354.1 |
| YN1 | 2019 | Yak | China | MH741353.1 |
| YN2 | 2019 | Yak | China | MH741352.1 |
| YN3 | 2019 | Yak | China | MH741351.1 |
| YN4 | 2019 | Yak | China | MH741350.1 |
| YN5 | 2019 | Yak | China | MH741349.1 |
| YN6 | 2019 | Yak | China | MH741348.1 |
| YN7 | 2019 | Yak | China | MH741347.1 |
| YN8 | 2019 | Yak | China | MH741346.1 |
| YN9 | 2019 | Yak | China | MH741345.1 |
| YN10 | 2019 | Yak | China | MH741344.1 |
| YAK/HY24/CH/2017 | 2022 | Yak | China | MH810163.1 |
