# Supplementary figures and images for: Specific and sensitive detection of bovine coronavirus using CRISPR-Cas13a combined with RT-RAA technology
Source: Front Vet Sci. 2025 Jan 7;11:1473674. doi: 10.3389/fvets.2024.1473674 (PMC11749252; doi:10.3389/fvets.2024.1473674)

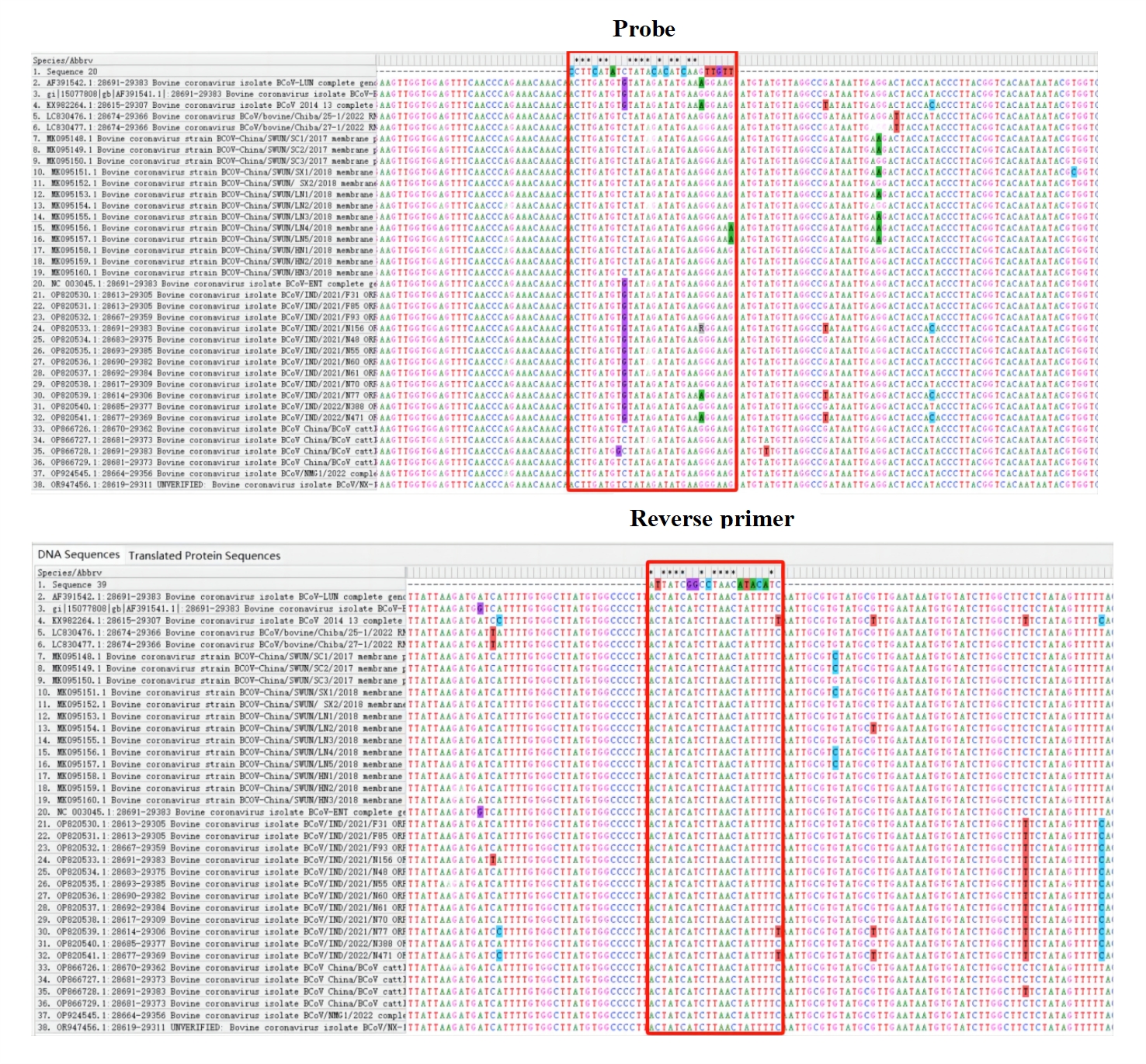

Supplement: Supplementary file 2 [file Image_1.JPEG]

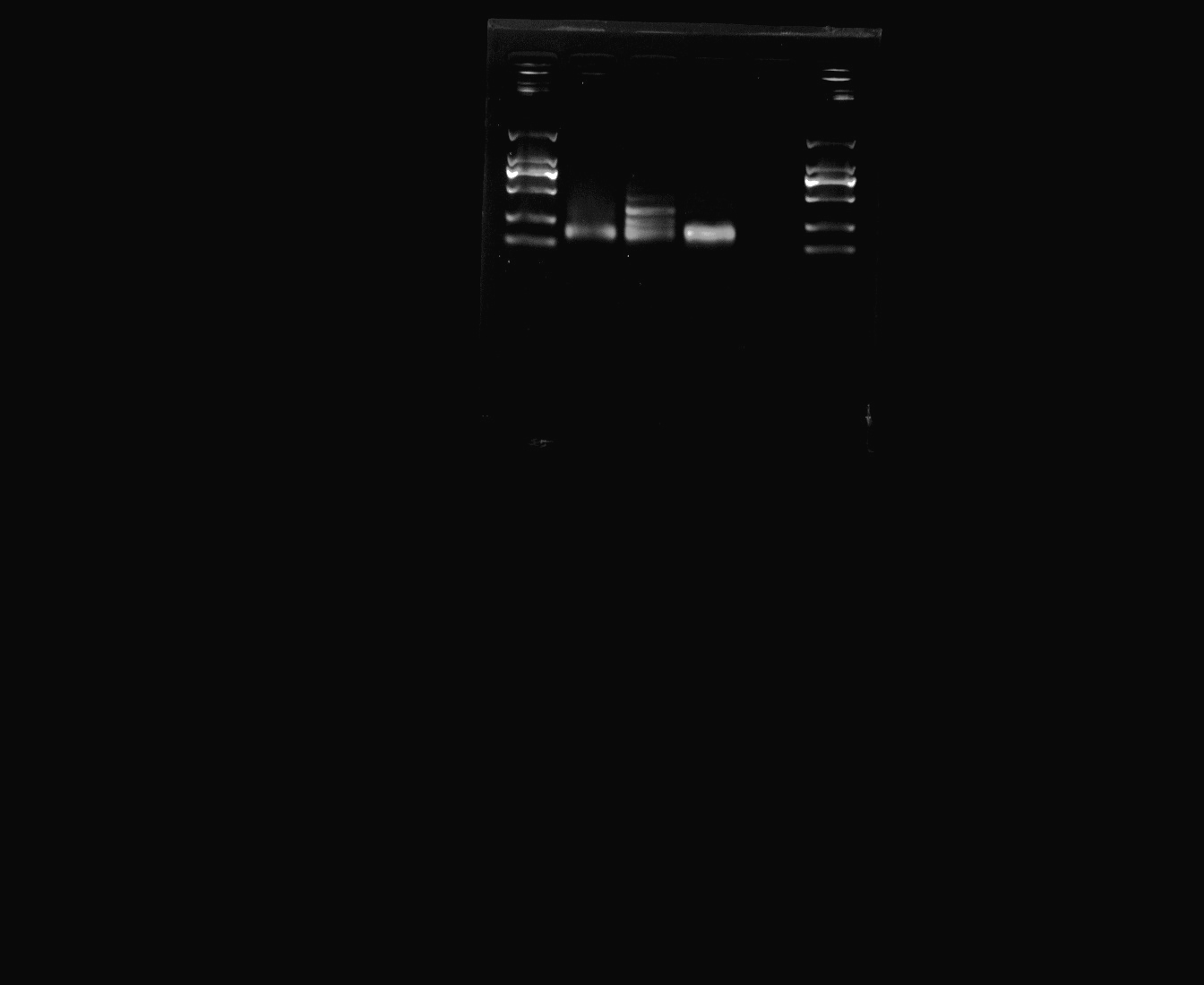

Supplement: Supplementary file 3 [file Image_2.JPEG]

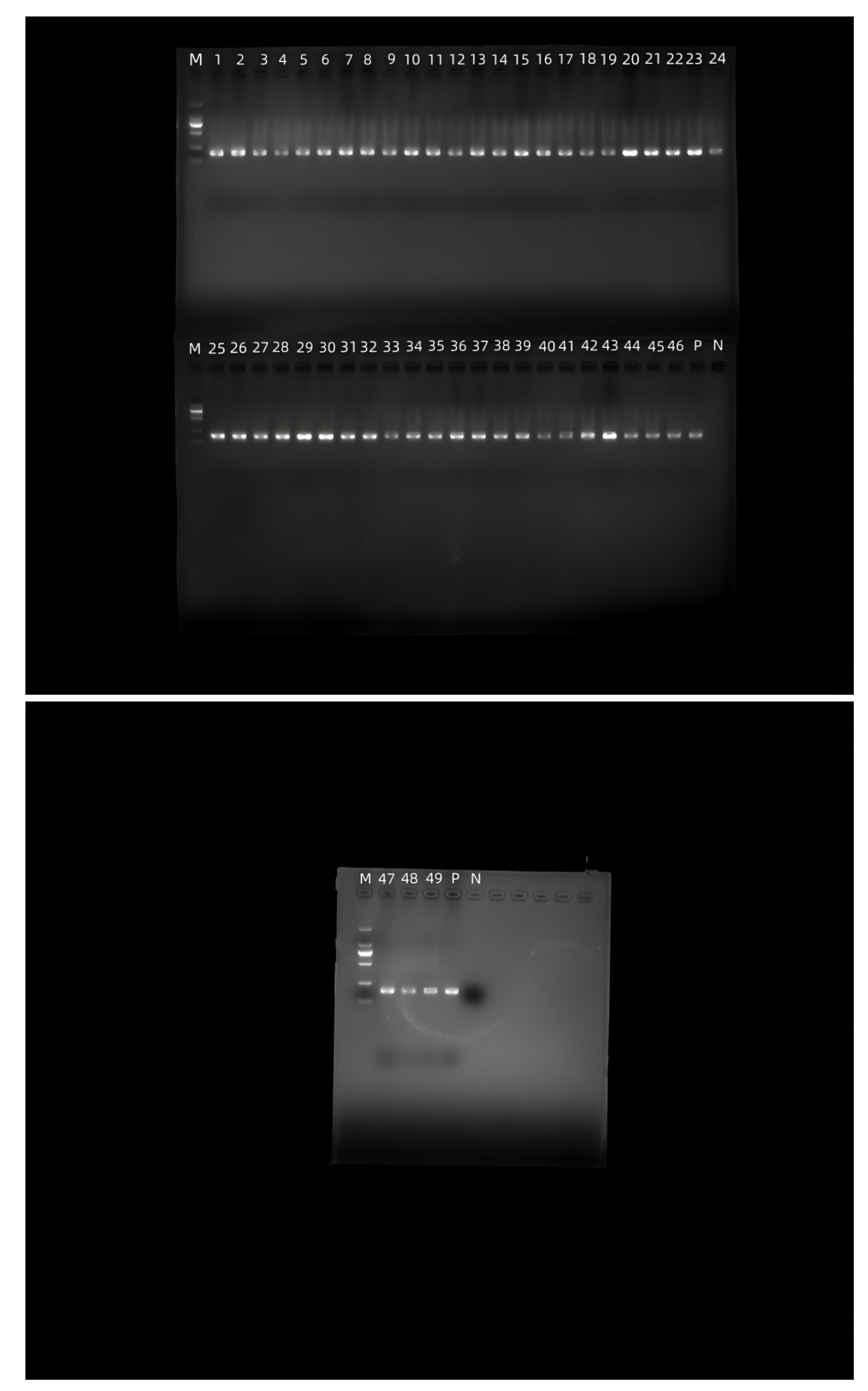

Supplement: Supplementary file 4 [file Image_3.PNG]
